# Risk factors for acute compartment syndrome of the leg associated with tibial diaphyseal fractures in adults

**DOI:** 10.1007/s10195-014-0330-y

**Published:** 2014-12-28

**Authors:** Babak Shadgan, Gavin Pereira, Matthew Menon, Siavash Jafari, W. Darlene Reid, Peter J. O’Brien

**Affiliations:** 1 Trauma Orthopaedic Division, Department of Orthopaedics, University of British Columbia, #110-828W 10th Ave, Vancouver, BC V5Z 1L8 Canada; 2 University Hospital Coventry and Warwickshire, Clifford Bridge Road, Coventry, CV2 2DX UK; 3 Division of Orthopaedic Surgery, University of Alberta, 10150-121 Street, Edmonton, AB T5N 1K4 Canada; 4 School of Population and Public Health, Faculty of Medicine, University of British Columbia, 2206 East Mall, Vancouver, BC V6T 1Z3 Canada; 5 Department of Physical Therapy, University of Toronto, 160-500 University Avenue, Toronto, ON M5G 1V7 Canada; 6 5440-ICORD, Blusson Spinal Cord Centre, 818 West 10th Avenue, Vancouver, BC V5Z-1M9 Canada

**Keywords:** Tibia fracture, Compartment syndrome, Fracture fixation intramedullary

## Abstract

**Background:**

We sought to examine the occurrence of acute compartment syndrome (ACS) in the cohort of patients with tibial diaphyseal fractures and to detect associated risk factors that could predict this occurrence.

**Materials and methods:**

A total of 1,125 patients with tibial diaphyseal fractures that were treated in our centre were included into this retrospective cohort study. All patients were treated with surgical fixation. Among them some were complicated by ACS of the leg. Age, gender, year and mechanism of injury, injury severity score (ISS), fracture characteristics and classifications and the type of fixation, as well as ACS characteristics in affected patients were studied.

**Results:**

Of the cohort of patients 772 (69 %) were male (mean age 39.60 ± 15.97 years) and the rest were women (mean age 45.08 ± 19.04 years). ACS of the leg occurred in 87 (7.73 %) of all tibial diaphyseal fractures. The mean age of those patients that developed ACS (33.08 ± 12.8) was significantly lower than those who did not develop it (42.01 ± 17.3, *P* < 0.001). No significant difference in incidence of ACS was found in open versus closed fractures, between anatomic sites and following IM nailing (*P* = 0.67). Increasing pain was the most common symptom in 71 % of cases with ACS.

**Conclusions:**

We found that younger patients are definitely at a significantly higher risk of ACS following acute tibial diaphyseal fractures. Male gender, open fracture and IM nailing were not risk factors for ACS of the leg associated with tibial diaphyseal fractures in adults.

**Level of evidence:**

Level IV.

## Introduction

Fracture of the tibia is the most common long-bone fracture worldwide [1]. Acute compartment syndrome (ACS) is considered to be one of the most serious complications of tibial fractures, and failure to diagnose and treat it in time can lead to catastrophic consequences that are devastating to patients, surgeons and health care providers. Giannoudis et al. [2] have shown that patients who sustained a tibial fracture followed by an ACS, performed worse on the EuroQol score than those who had uncomplicated fractures. Delayed or missed diagnosis of ACS following tibial shaft fracture negatively affects the health care team as well. In addition to the psychological stress for health care givers associated with poor patient outcome, the average indemnity paid for missed ACS is high and the rate of successful defence of cases is lower than with other orthopaedic medico legal cases [3, 4]. The cost of ACS is significant, resulting in prolonged hospital stays and charges that are more than doubled in patients with tibial fractures affected by ACS [5]. Physicians treating patients with traumatic injuries are normally aware of ACS. However, due to the low incidence of this condition, a high index of suspicion is usually required to initiate one’s thought processes towards making a diagnosis of ACS. There has been no large-scale study on the epidemiology of lower leg compartment syndrome so far. It is therefore difficult to appreciate the burden of this problem.

The purpose of this study, therefore, was to examine the relationship between the development of ACS in the cohort of patients presenting to our hospital with tibial diaphyseal fractures and specific demographic, injury, and operative characteristics that could predict this occurrence. A thorough understanding of these risk factors and their relative influence on development of lower leg ACS may provide better insight into the recognition of high risk individuals, which is critical in an effort to optimize patient outcomes.

## Materials and methods

This retrospective cohort study was conducted at a level-one trauma centre attended by five full-time orthopaedic trauma surgeons. All tibial diaphyseal fractures that were treated between January 1997 and December 2011 were retrieved from the orthopaedic trauma prospective data base. Patients developing ACS in this group were identified and relevant information on demographics and risk factors were collected.

ACS of the lower leg was defined for the purpose of this study as being an acute event following a tibial diaphyseal fracture diagnosed by clinical signs and symptoms and, where necessary, by intra-compartmental pressure measurements, using a handheld intra-compartmental pressure monitoring system (Stryker Surgical, Kalamazoo, MI), but confirmed at fasciotomy and entered prospectively into the data base as ‘Acute Compartment Syndrome’. Patients who were treated with non-surgical fixation methods or primary amputation, or patients who had open fractures requiring vascular repair or intra-articular fractures, were excluded.

Information regarding gender, age, year of injury, mechanism of injury, injury severity score (ISS), fracture side, state of skin/soft tissue injury [6], site of fracture along the tibial shaft and method of fixation were abstracted from the data base. For the purpose of this study, mechanism of injury was subdivided into twisting, fall, direct blow, crushing injury, vehicle accident, bicycle accident, motorcycle accident, and pedestrian vs motor vehicle accident. The site of fracture was classified as being in the proximal, middle or distal third of the tibial diaphysis. When the fracture crossed 2 zones, it was entered as such. Extensive fractures were those that crossed all 3 zones. Methods of surgical fixation were classified into intramedullary (IM) nailing (dynamic locking nail, static locking nail, unlocked nail) and non-IM nailing (screw, plate, external fixator) methods. The choice of fixation was based on the pattern of fracture, the soft tissue involvement, and the general condition of the patient before and after the injury as well as surgeon’s preference. The mean lengths of hospital stays of patients with and without ACS were also compared.

In addition we undertook a chart review of the 87 cases of ACS and recorded levels of consciousness, clinical symptoms and signs, intra-compartmental pressure measures as well as time interval between occurrence of tibial fracture and surgical fixation to fasciotomy.

The incidence of ACS following tibia diaphyseal fractures was determined from the data. Data on demographics, type of trauma, side of tibia fracture, year of fracture, open vs closed fracture, anatomical classification of the fracture, fracture pattern, mechanism of fracture, method of internal fixation, clinical symptoms at admission, and intra-compartmental pressure measures of all cases were collected. Student’s *t*-test was used to compare the means of two groups. Pearson’s chi square test or Fisher’s exact tests were used to compare categorical variables. Relative risks and 95 % confidence interval (95 %CI) were calculated to assess the association between potential risks factors and development of ACS. Statistical significance at 5 % was selected in this study and the relative risk (RR) with 95 % confidence interval (CI) is reported where appropriate. STATA statistical software (StataCorp 2011. Stata Statistical Software: release 12. College Station, TX: StataCorp LP) was used for data analysis.

## Results

For the 14-year period 1997–2011, inclusive, 1,125 tibial fractures were identified in 1,100 patients. Table 1 lists the characteristics of patients with tibia fractures included in this study. ACS of the leg occurred as an immediate or early complication in 87 limbs, 7.73 % of all tibia fractures. Henceforth in this paper, all statistics will refer to the tibial fractures and not patients. Characteristics of patients with tibial fractures and those who developed ACS are summarized in Tables 1 and 2, respectively.

**Table 1:** Characteristics of the patients included in the study

		*N* (1,125)	%
Age mean	41.32 (±17.2)		
Gender	Male	772	69
	Female	353	31
Trauma type	Single injury	662	58.84
	Multiple injury	179	15.91
	Multiple system trauma	284	25.24
Side of tibia fracture	Right	553	49.16
	Left	572	50.84
Skin	Close fracture	776	68.98
	Open fracture	349	31.02
Fracture classification	Proximal/3	147	13.07
	Middle/3	632	69.24
	Distal/3	265	23.56
	Extensive	81	7.2
Mechanism of fracture	Fall	399	35.47
	Pedestrian vs motor vehicle accident	305	27.11
	Motorcycle accident	188	16.71
	Twisting injury	80	7.11
	Direct blow	72	6.4
	Crushing injury	40	3.56
	Bicycle accident	23	2.04
	Motorized accident	18	1.6
Internal fixation method	Screw	21	1.87
	Plate	186	16.53
	External fixator	3	0.27
	Dynamic locking nail	22	1.96
	Static locking nail	886	78.76
	Unlocked nail	7	0.62
Early local complication	None	1,014	90.13
	Compartment syndrome	87	7.73
	Reduction/fixation failure	13	1.16
	Wound infection	7	0.62
	Neurovascular loss	4	0.36

**Table 2:** Characteristics of patients with tibial fractures who developed ACS

		*N*	%
Age mean	33.08 (±12.8)		
Gender	Male	66	76
	Female	21	24
Skin	Closed	66	75.86
	Open	21	24.14
Fracture classification	Proximal/3	11	12.64
	Middle/3	43	49.43
	Distal/3	25	28.74
	Extensive	8	9.2
Clinical signs and symptoms	Severe pain	31	35.23
	Paresthesia	7	7.95
	Motor weakness	4	4.55
	Unconscious	6	6.82
	Pain and paresthesia	17	19.32
	Pain and paresthesia and motor weakness	14	15.91
	Paresthesia + motor weakness	8	9.09
	Pressure	1	1.14
Mechanism of injury	Fall	26	29.89
	Pedestrian vs motor vehicle	22	25.29
	Motor accident	19	21.84
	Twisting injury	6	6.9
	Blow	6	6.9
	Crushing injury	5	5.75
	Motorized accident	2	2.3
	Bicycle	1	1.15
Fracture pattern	Comminuted	23	26.14
	Oblique	17	19.32
	Segmental	8	9.09
	Spiral	16	18.18
	Transverse	24	27.27
Involved compartments	Anterior	37	42.53
	Lateral	4	4.6
	Posterior	3	3.45
	Not specified	19	21.84
	Anterior + lateral	13	14.94
	All	11	12.64

The mean age of all participants was 41.32 (±17.2) with a range of 16–99 years. Male patients were overall younger than female patients with a mean age of 39.60 (±15.97) compared with 45.08 (±19.04) for female patients, and this difference was statistically significant (*t* = 5.0143; *df* = 1,123, *P* < 0.0001). The mean age of those patients who developed ACS was 33.08 (±12.8), which was much lower than the mean age of patients who did not develop ACS (42.01 ± 17.3) and this difference was statistically significant (*P* < 0.001). Of the 1,125 tibial fractures, 772 were in males and 353 in females. Sixty-six out of 772 men (8.55 %) and 21 out of 353 women (5.95 %) developed ACS. Male gender was found to be a risk factor for development of ACS (RR = 1.11; 95 %CI: 0.98–1.26) but this risk was not statistically significant (Pearson’s *χ*^*2*^ = 2.2971, *df* = 1, *P* = 0.130) (Table 3).

**Table 3:** Comparison of patients with ACS with those without ACS

		Tibial fracture no ACS	Tibial fracture with ACS	Test statistics	*P* value
Age		42.01 (17.3)	33.08 (12.8)	*t* = 4.7037; *df* = 1123	<0.001
Gender	Male	706	66	*χ*^*2*^ = 2.29; *df* = 1	0.13
	Female	332	21		
Skin	Closed	710	66	*χ*^*2*^ = 2.0884; *df* = 1	0.148
	Open	328	21		
Fracture classification	Proximal/3	136	11	*χ*^*2*^ = 2.3738; *df* = 3	0.499
	Middle/3	589	43		
	Distal/3	240	25		
	Extensive	73	8		
Trauma type	Single injury	608	54	*χ*^*2*^ = 1.0464; *df* = 2	0.593
	Multiple system trauma	266	18		
	Multiple injury	164	15		
Fracture side	Left	529	43	*χ*^*2*^ = 0.0760; *df* = 1	0.783
	Right	509	44		
Fixation method Mechanism of injury	Static locking nail	812	74	*χ*^*2*^ = 3.1695; *df* = 5	0.674
	Plate	176	10		
	Dynamic locking nail	21	1		
	Screw	20	1		
	Unlocked nail	6	1		
	External fixator	3	0		
	Fall	399	26	*χ*^*2*^ = 4.4011; *df* = 7	0.733
	Pedestrian vs motor vehicle	305	22		
	Motor accident	188	19		
	Twisting injury	80	6		
	Blow	72	6		
	Crushing injury	40	5		
	Motorized accident	18	2		
	Bicycle	23	1		
ISS	01–10	802	76	*χ*^*2*^ = 4.8735; *df* = 3;	0.181
	11–20	109	5		
	21–30	74	4		
	>30	53	2		

In this study we did not find a significant relationship between type of fracture (open vs closed), anatomical site of tibia fracture, type of trauma, fixation method, or mechanism of injury and development of ACS. Although the relative risk of development of ACS was lower among patients with open fractures (RR = 0.76; 95 %CI: 0.52–1.12), this relationship was not statistically significant (χ^*2*^ = 2.0884; *df* = 1; *P* = 0.148). Among both groups, the distribution of the ISS was heavily influenced by the number of subjects with ISS 9. No difference could be found between these groups though.

**Fig. 1:**
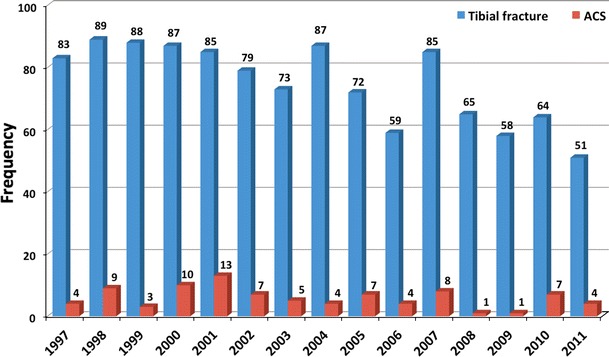
Frequency distribution of tibial fractures and ACSs during the study period

The number of tibial fractures admitted to our hospital decreased over the 14-year period of observation. However, the incidence of ACS following tibial fractures did not show a significant decline (Fig. 1).

A retrospective review of the charts showed that 7 % of patients with ACS had an altered level of consciousness. The clinical features in the awake patients with ACS included increasing pain in 70 %, paresthesia in 52 %, motor weakness in 29 % and tense swelling of the calf in 1 %. Thirty-five percent of patients with ACS had pain as their only symptom, 8 % had only parasthesia, 4 % had only motor weakness and 1 % had only tense calf.

Out of 87 patients with ACS, 23 patients underwent fasciotomy at the time of fracture fixation, while 64 patients received fasciotomy as a second surgical procedure after initial fixation. The mean time interval between occurrence of tibia fracture and fasciotomy was 30.10 (±23.72) h. The mean time interval between surgical fixation of fracture and fasciotomy was 16.27 (22.58) h. The mean length of hospitalization of those patients who developed ACS was 14.88 (±11.80) days, which was higher than the mean length of hospitalization of patients who did not develop ACS (12.26 ± 10.28) and this difference was statistically significant (*P* = 0.03).

Using an intra-compartmental pressure monitoring instrument, compartment pressures were measured in 60 out of 87 patients with ACS and 92 % had an absolute reading of greater than 30 mmHg. This raised intra-compartmental pressure occurred in the anterior compartment in 87 %, lateral compartment in 35 % and posterior compartments in 37 % of cases. All patients with ACS have positive intra-operative findings.

## Discussion

Over the last four decades, much research has been published with regards to the pathophysiology [7–11], diagnosis [12–14], monitoring [13–15], and treatment of ACS [16–20]. However, there is little literature regarding the epidemiology of lower leg ACS and its associated risks factors [21, 22].

This epidemiological study on lower leg ACS is a retrospective cohort study, but we believe that the data quality is good, as the data was entered prospectively by the treating surgeons themselves and the data base was managed by a dedicated research staff. Our results are compared with other studies in Table 4.

**Table 4:** Summary of studies on ACS in tibial diaphyseal fractures

Author	Number of subjects	Number of ACS	Incidence (%)
DeLee (open) [38]	104	6	6
Blick SS [24]	198	18	9
McQueen (open) [33]	67	1	1.5
McQueen [23]	1,349	59	4.3
Mullett H [34]	626	17	2.7
Ogunlusi JD [39]	52	3	5.7
Park S [21]	173	14	8
Wind T [26]	626	34	5.4
This study	1,125	87	7.7

Acute compartment syndrome from any cause occurs most commonly in the lower leg and most often follows a fracture of the tibia. McQueen et al. [23] reported in their epidemiological study that 36 % of all compartment syndromes occurred in association with a tibial shaft fracture. They found that the occurrence of ACS following tibial fractures was 4.3 %. In North America, the prevalence has been reported in the range of 5.4–11 % [24–26]. There has been no other epidemiological study on compartment syndromes in a particular population.

In our study, over a 14-year period, we found that the average incidence of ACS in tibial fractures was 7.73 %. In spite of significant improvement in management of fractures and their related complications during recent years, the incidence of ACS following tibial fracture is not significantly reduced. This is likely to be multifactorial. One reason could be the increased trend to internally fix tibial shaft fractures in more recent times. In addition, continuous improvement in the survivability of patients with multiple injuries might lead to a larger number of patients surviving with ACS when they may have died before.

The popular belief is that ACS is more likely to occur in young males. In our study we found that women were just as likely as men to get ACS following their tibial fractures. However, we also found that ACS occurred more readily in younger patients. This we think is due to younger people having larger muscle bulk within a tight fascia with very little room to expand before the intra-compartmental pressure rises. Park et al. [21] also found age as a risk factor. They suggested that the young male is not only likely to have a larger muscle bulk, but the fascia and the inter-muscular septa are likely to be thicker, due to a larger collagen content. This can cause the pressure to rise rapidly within a compartment with a small increase in volume. We would agree with their hypothesis; however, we did not find a higher incidence of ACS in males. Therefore, we do not assume gender to be risk factor for ACS following tibial fracture.

Many physicians believe that high-energy trauma should be a risk factor for ACS. The perceived wisdom is that the soft tissue damage that occurs with a high energy transfer is likely to produce more necrosis, hypoxia, lactic acidosis, capillary leak and more interstitial fluid collection, leading to swelling of the compartment. However, this was not borne out in our study. This observation is similar to what Court-Brown et al. [27] reported.

There is no doubt that multiple injuries that affect a number of anatomical sites have a profound effect on the homeostasis of the body and the ensuing “chemical storm”: systemic inflammatory response syndrome (SIRS) vs compensatory anti-inflammatory response syndrome (CARS) and endothelial damage is linked with occurrence of ACS [28]. However, ACS could be both one of the triggering factors for SIRS or indeed an effect of the endothelial damage and subsequent capillary leak. We assumed that those patients with a higher ISS score were more likely to get ACS. Polytrauma patients with high ISS scores are likely to be hypotensive and, theoretically, ACS is likely to occur at a lower compartment pressure when the diastolic is lower: causing a lowered perfusion pressure (ΔP) [13]. In addition, polytrauma patients are often aggressively resuscitated with high volumes of fluid that can then enter the extravascular space in injured compartments and increases the intra-compartmental pressure. This was not seen in our study. In the study by Park et al., the arterial blood pressure of patients at admission was recorded and the authors found no correlation between hypotension and the incidence of ACS.

Although there is a risk of ACS with any type of tibia fracture, an open fracture is anecdotally considered to have de facto decompressed the compartments, and is therefore unlikely to cause an ACS. The auto decompression phenomenon that occurs with a high-grade open tibia fracture is hypothesized to cause an effect similar to a fasciotomy [23, 27]. Our study showed that ACS is just as likely to occur in open fractures as it is in closed fractures. These results are similar to those found by Park et al. [21].

We looked at the site of the fracture as a potential risk factor because the tibial shaft has various muscle attachments and varying bulk of muscle at different levels. The gastro-soleus complex is bulkier more proximally than distally. There is also less muscle and more tendinous structures more distally. If one considers that ACS can occur from bleeding alone, (as opposed to ischemic swelling), then it should be more common in more proximal fractures. In contrast, the peroneus tertius, whose muscle belly is alongside the distal third of the tibia, has a single arterial supply. It is conceivable that any fractures in the distal third of the tibia, which might damage the only blood supply to the muscle could cause ischemia, capillary leak and swelling followed by ACS. In this study, we could not show that the site of the fracture made any difference to the occurrence of ACS. Fractures that are more extensive along the length of the bone suggest a higher energy transfer. Similar to our findings for mechanism of injury, we did not find any correlation between ‘extensive’ fractures and ACS.

There are reports confirming a significant correlation between intramedullary nailing and ACS development [29–34]. These reports argue that: (1) the incidence of ACS in open reduction and internal fixation is likely to be lower due to the pari passu decompression of the compartment and evacuation of the fracture haematoma; (2) intramedullary nailing is known to increase the compartment pressures during reaming as well as insertion of the nail; and (3) the position of the limb during the procedure has shown to change the compartment pressures. Patients put on traction tables with traction applied to the limb during nailing have raised intra-compartmental pressures [35]. In this study we did not find a significantly higher rate of ACS development in those who were treated by intramedullary nailing, though.

Our data indicated that the length of hospital stays of patients with tibial fractures who were affected by ACS was higher than those without ACS. This finding supports previous reports of increased hospital stays up to 3 times longer in patients with ACS compared with uncomplicated tibia fractures [5]. This difference is likely due to at least two additional surgical procedures for wound closure in patients who are affected by this complication.

In our study, increasing leg pain was the main clinical symptom in patients with ACS, followed by paresthesia and motor weakness of leg muscles. Furthermore, the anterior compartment was the most involved compartment of the leg and absolute measures of intra-compartmental pressures of leg compartments were higher than 30 mmHg in 92 % of patients. These findings are in accordance with the literature [36].

There are shortcomings in this study. We did not collect data regarding some other potential risk factors such as a history of smoking prior to the injury. We were also not able to distinguish between cases of ACS that were diagnosed on the basis of a fasciotomy alone. O’Toole et al. [37] showed that within the same institution, the fasciotomy rate among surgeons varied. We did not analyse this. We also did not have records of patient consciousness levels in our data base, and hence we are unable to say whether ACS was more likely to occur in unconscious patients than conscious patients (who can complain of severe pain). However, we believe that consciousness levels are not variables that would directly influence the occurrence of ACS and therefore are not a direct risk factor.

This is one of the largest studies examining the possible risk factors that influence the occurrence of ACS in tibial diaphyseal fractures treated by surgical fixation. We found that younger patients are definitely at a significantly higher risk of ACS. Gender, mechanism of injury, Gustillo and anatomical classification, ISS and intramedullary nailing of tibial fracture did not influence ACS.
